# Supplementary material for: Single Cell Transcriptomic and Chromatin Profiles Suggest Layer Vb Is the Only Layer With Shared Excitatory Cell Types in the Medial and Lateral Entorhinal Cortex
Source: Front Neural Circuits. 2022 Jan 26;15:806154. doi: 10.3389/fncir.2021.806154 (PMC8826650; doi:10.3389/fncir.2021.806154)
Supplement: Supplementary file 14 [file Data_Sheet_1.ZIP › MEC_Sup_vs_LEC_Sup_Motifs/homerResults/motif2.similar.html]

motif2

## Information for motif2

A
G
C
T
A
G
C
T
G
T
A
C
A
T
G
C
T
C
G
A
C
A
G
T
G
T
C
A
A
G
C
T
A
T
C
G
A
C
T
G
A
G
C
T
G
A
C
T
  
Reverse Opposite:  

C
T
G
A
T
C
G
A
A
G
T
C
A
T
G
C
C
T
G
A
C
A
G
T
G
T
C
A
A
G
C
T
A
T
C
G
A
C
T
G
T
C
G
A
T
C
G
A
  

|  |  |
| --- | --- |
| p-value: | 1e-142 |
| log p-value: | -3.292e+02 |
| Information Content per bp: | 1.781 |
| Number of Target Sequences with motif | 4592.0 |
| Percentage of Target Sequences with motif | 21.79% |
| Number of Background Sequences with motif | 1850.2 |
| Percentage of Background Sequences with motif | 15.16% |
| Average Position of motif in Targets | 307.6 +/- 229.6bp |
| Average Position of motif in Background | 303.1 +/- 251.1bp |
| Strand Bias (log2 ratio + to - strand density) | -0.0 |
| Multiplicity (# of sites on avg that occur together) | 1.14 |
| Motif File: | file (matrix) reverse opposite |

### Similar de novo motifs found

|  |  |  |  |  |  |  |  |
| --- | --- | --- | --- | --- | --- | --- | --- |
| Rank | Match Score | Redundant Motif | P-value | log P-value | % of Targets | % of Background | Motif file |
| 1 | 0.761 | A G T C C G T A A C G T C G T A A C G T C T A G A C G T G C T A | 1e-126 | -291.769408 | 39.90% | 32.04% | motif file (matrix) |
| 2 | 0.685 | G T A C G T A C G T C A C A T G C T G A G A T C A T C G T C A G | 1e-72 | -167.115419 | 49.21% | 43.02% | motif file (matrix) |
